# Nationwide high prevalence of CTX-M and an increase of CTX-M-55 in Escherichia coli isolated from patients with community-onset infections in Chinese county hospitals

**DOI:** 10.1186/s12879-014-0659-0

**Published:** 2014-12-03

**Authors:** Jing Zhang, Beiwen Zheng, Lina Zhao, Zeqing Wei, Jinru Ji, Lanjuan Li, Yonghong Xiao

**Affiliations:** Collaborative Innovation Center for Diagnosis and Treatment of Infectious Diseases, State Key Laboratory for Diagnosis and Treatment of Infectious Disease, The First Affiliated Hospital, College of Medicine, Zhejiang University, Hangzhou, 310003 China

## Abstract

**Background:**

In order to investigate the epidemiology, molecular characteristics, and distribution of extended-spectrum β-lactamase (ESBL)- and AmpC-producing *Escherichia coli* from community-onset infections in Chinese county hospitals.

**Methods:**

*E. coli* isolates were collected from patients with community-onset infections in 30 county hospitals. ESBL activity was confirmed by double-disc diffusion. Genetic confirmation and molecular typing of ESBL- and AmpC-producing isolates was determined by PCR and DNA sequencing. ESBL-positive isolates were further characterised by multi-locus sequence typing.

**Results:**

Of 550 *E. coli* isolates, 256 (46.5%) carried ESBL genes and all were of the CTX-M type. The prevalence of ESBL-producing strains varied from 30.2% to 57.0% across different regions of China. Overall, 12 *bla*_CTX-M_ subtypes were detected; the most abundant were *bla*_CTX-M-14_ (163/256 isolates, 64.5%), *bla*_CTX-M-55_(47/256, 18.4%), and *bla*_CTX-M-15_ (31/256, 12.1%). CMY-2-like AmpC β-lactamases were detected in 11 strains, three of which co-existed with *bla*_CTX-M_. A total of 64 sequence types (STs) were detected in 256 ESBL-producing strains, including nine that were new. ST131 was the most abundant type (27 isolates, 12.7%), followed by ST69 (14 isolates, 6.6%), ST405 (14 isolates, 6.6%), and ST38 (12 isolates, 5.6%).

**Conclusions:**

This study revealed that the widespread prevalence of ESBLs among outpatient infections has reached a high level in county hospitals. The CTX-M genotype was most dominant, comprising a variety of subtypes. This is the first time the incidence of CTX-M-55 has exceeded that of CTX-M-15 in China. No predominant ST was detected, suggesting that ESBL-producing *E. coli* strains originate in different clones*.*

**Electronic supplementary material:**

The online version of this article (doi:10.1186/s12879-014-0659-0) contains supplementary material, which is available to authorized users.

## Background

The production of extended-spectrum β-lactamases (ESBLs) is the main mechanism of resistance to β-lactam antibiotics in *E. coli.* ESBLs hydrolyse penicillins, cephalosporins, and aztreonam; however, they are inhibited by clavulanic acid, sulbactam, and tazobactam [1],[2]. The enzymes TEM and SHV were previously recognised as the main ESBLs. However, CTX-Ms have recently become more prominent, and are now considered to be the most prevalent β-lactamases found in clinical isolates of *E. coli* globally [3],[4]. A nationwide survey of the United States during 2009–2010 revealed that 91% of ESBL-producing *E. coli* strains carried CTX-M-type genes [5]. CTX-M-type ESBLs are also dominant in European and Asian countries [4],[6], and in China they are the most common ESBLs found in Gram-negative bacteria isolated from tertiary hospitals [7]-[9]. ESBL-producing *E. coli* strains are a major cause of community-associated infections [5],[10], and their spread has been well-documented in Europe and North America [5],[11]-[15]. Strains of ESBL-producing *E. coli* have been recorded in other regions of the world, including Oceania, Asia, and South America [10],[16]-[18]. Several studies have characterised ESBL-producing *Enterobacteriaceae* in Chinese tertiary hospitals [7],[19],[20]. A national monitoring program of antimicrobial resistance in *E. coli* caused bacteriaemia (mostly nosocomial infections) in Chinese tertiary hospitals reported an increase in ESBL-producing *E. coli* from less than 20% to 72.2% between 2000 and 2011 [21]-[23]. Another survey of Chinese tertiary hospitals reported that the incidence of ESBL-producing *E. coli* among community-associated intra-abdominal infections increased from 19.1% in 2002–2003 to 61.6% in 2010–2011 [20]. None of these studies focused on bacteria isolated from outpatients in primary and non-central city hospitals. In light of the increasing importance of ESBL-producing *E. coli* within communities, it is important to understand the pattern of antibiotic resistance and the prevalence of ESBLs in bacterial isolates from primary and non-central city healthcare institutions that provide medical services to patients in China.

Most Chinese people live in rural areas or small to medium-sized cities, and seek their healthcare at county-level hospitals, which play an important role in China. The high global prevalence of ESBL-producing *E. coli* strains among community-onset infections warrants an urgent need to investigate the characteristics of antimicrobial resistance in China. This study focuses on community-onset infections in patients at selected hospitals in seven regions of China. The purpose was to investigate the antimicrobial resistance, the incidence of ESBLs and their genetic types, and the molecular characteristics of *E. coli* strains isolated from patients, with the intention of developing strategies to control and prevent antimicrobial resistance.

## Methods

### Collection of clinical isolates

Thirty county hospitals were selected for this investigation (see Additional file 1), which took place between August 2010 and August 2011, and in total 550 *E.coli* were collected. These hospitals are located in 11 different provinces and represent seven geographic regions of China. In China, hospitals are categorized by the level of service provision, size, medical technology, medical equipment, and management and medical quality into 3 subsidiary levels, primary hospitals, secondary hospitals, tertiary hospitals. All the county hospitals included in this study are secondary hospitals. Patients were selected for this study using the following criteria: 1) they attended an outpatient clinic or emergency department and were diagnosed with a bacterial infection less than 48 hours after they were hospitalised; 2) they were not admitted to hospital within 90 days prior to diagnosis; 3) they did not have long-term indwelling catheters; and 4) and they had not received antibacterial therapy or had received less than 72 hours of antibacterial therapy prior to seeing a doctor. Bacterial strains were isolated from clinical specimens (urine, blood, sputum, abscesses, and secretions) and identified using API20 (bioMérieux, Durham, NC, USA). Samples were collected as part of routine care from outpatients; pathogens were isolated by standard microbiological methods by the microbiologists of the participant county hospitals. All the pure cultures were frozen at -80°C and shipped to our laboratory for definite identification and further analysis. The identities of all isolates were ratified using Matrix-assisted laser desorption ionisation-time of flight mass spectrometry (MALDI-TOF) as described previously [24].

### Antimicrobial susceptibility testing

Antimicrobial susceptibility was tested using the standard agar dilution method recommended by the Clinical and Laboratory Standards Institute guidelines (M100-S21) [25]. The breakpoint of biapenem was based on the recommended point of imipenem by CLSI. The following antimicrobial agents were tested: ampicillin, piperacillin, cefazolin, cefuroxime, ceftazidime, ceftriaxone, cefepime, ampicillin-sulbactam, piperacillin-tazobactam, cefoxitin, biapenem, imipenem, meropenem, amikacin, gentamicin, ciprofloxacin, levofloxacin, and fosfomycin (Chinese National Institute for the Control of Pharmaceutical and Biological Products). ESBL activity was determined by the double-disc diffusion method using ceftazidime (30 μg), ceftazidime plus clavulanate (30/10 μg) discs and cefotaxime (30 μg), and cefotaxime plus clavulanic acid (30/10 μg) discs (Oxoid Limited, UK) on Mueller-Hinton agar (Oxoid Limited, UK) [25]. The results of antimicrobial susceptibility testing were analyzed by WHONET5.6. The reference strains used for this study were *E. coli* ATCC 25922 and *Klebsiella pneumoniae* ATCC 700603.

### Detection of β-lactamase genes

PCR amplification was used to detect *bla*_CTX-M_, *bla*_SHV_, *bla*_TEM_, *bla*_OXA_, *bla*_VEB_, *bla*_PER_, and *bla*_GES_, *bla*_KPC_ and the AmpC β-lactamase genes *bla*_CMY-1_, *bla*_CMY-2_, and *bla*_DHA_. *bla*_CTX-M_ group-specific primers for CTX-M-1, CTX-M-2, CTX-M-8 and CTX-M-9 groups were used to detect of *bla*_CTX-M_ genes, and three pair of group specific primers (OXA-1, OXA-2, OXA-10) for detecting *bla*_OXA_. Genomic DNA of clinical isolates was prepared using a modified boiling method [26] and this DNA was used as the template for PCR amplification. PCR products were separated by electrophoresis using a 1% agarose gel to identify the amplified DNA fragments. Forward and reverse sequencing reactions were performed on an ABI 3730 automated sequencer using ABI Prism BigDye Terminator version 3.1 cycle sequencing (Applied Biosystems, Foster City, CA, USA). Primers used for PCR detection and sequencing are listed in Table 1. Gene sequences were analysed online using BLAST (http://blast.ncbi.nlm.nih.gov/Blast.cgi) and identified using a β-lactamase database (http://www.lahey.org/Studies/).

**Table 1:** **Primers used for PCR amplification of bla genes**

PCR target	Primer	Sequence (5′–3′)	Fragment size, bp	Ref
*bla*_TEM_	TEM-F	CATTTCCGTGTCGCCCTTATTC	800	[27]
	TEM-R	CGTTCATCCATAGTTGCCTGAC		
*bla*_SHV_	SHV-F	GGTTATGCGTTATATTCGCC	867	[28]
	SHV-R	TTAGCGTTGCCAGTGCTC		
CTX-M-1 group	CTX-M-1F	CCCATGGTTAAAAAATCACTGC	942	[7]
	CTX-M-1R	CAGCGCTTTTGCCGTCTAAG		
CTX-M-2 group	CTX-M-2F	CGACGCTAC CCCTGC TAT T	552	[29]
	CTX-M-2R	CCAGCGTCAGATTTTTCAGG		
CTX-M-8 group	CTX-M-8F	AACR(A)CR(G)CAGACGCTCTAC	326	[27]
	CTX-M-8R	TCGAGCCGGAAGGTGTCAT		
CTX-M-9 group	CTX-M-9F	ATGGTGACAAAGAGAGTGCAAC	876	This study
	CTX-M-9R	TTACAGCCCTTCGGCGATGATT		
*bla*_OXA-1_	OXA-1F	GGCACCAGATTCAACTTTCAAG	814	[27]
	OXA-1R	GACCCCAAGTTTCCTGTAAGTG		
*bla*_OXA-2_	OXA-2F	GGCACCAGATTCAACTTTCAAG	702	[27]
	OXA-2R	GATTTGCTCCGTGGCCGAAA		
*bla*_OXA-10_	OXA-10F	ACGGCATTAGCTGGTTCAAT	720	This study
	OXA-10R	TGATTTTGGTGGGAATGGAT		
*bla*_GES_	GES-F	AGTCGGCTAGACCGGAAAG	399	[27]
	GES-R	TTTGTCCGTGCTCAGGAT		
*bla*_PER_	PER-F	GCTCCGATAATGAAAGCGT	520	[27]
	PER-R	TTCGGCTTGACTCGGCTGA		
*bla*_VEB_	VEB-F	CATTTCCCGATGCAAAGCGT	648	[27]
	VEB-R	CGAAGTTTCTTTGGACTCTG		
*bla*_CMY2_	CMY2-F	AACACACTGATTGCGTCTGA	1228	[30]
	CMY2-R	TCCTGGGCCTCATCGTCAGTTAT		
*bla*_CMY1_	CMY1-F	TATTAGAGCGGTTTAGGCTG	1456	[30]
	CMY1-R	AATGTACCGCCCTCTTTC		
*bla*_DHA_	DHA-F	TGATGGCACAGCAGGATATTC	997	[27]
	DHA-R	GCTTTGACTCTTTCGGTATTCG		
*bla*_KPC_	KPC-F	CATTCAAGGGCTTTCTTGCTGC	538	[27]
	KPC-R	ACGACGGCATAGTCATTTGC		

### Molecular typing of E. coli

To determine if ESBL genes were disseminated by clonal spread or horizontal gene transfer, isolates (n = 213) representing different regions were analysed by multi-locus sequence typing (MLST) following the guidelines found at http://mlst.warwick.ac.uk/mlst/dbs/Ecoli. Sequence typing of the housekeeping genes *adk*, *fumC*, *gyrB*, *icd*, *mdh*, *purA*, and *recA* was performed on all ESBL-producing *E. coli* isolates according to Wirth *et al*. [31]. Sequence types (STs) were confirmed by comparison with those in the database. A phylogenetic tree of STs detected in this study was constructed using Phylodendron (http://iubio.bio.indiana.edu/soft/molbio/java/apps/trees/). This tool uses the PHYLIP (a free package of programs for inferring phylogenies) suite of programs to generate trees from allelic profile data.

### Statistical analysis

Statistical significance was determined by *χ*^*2*^ analysis, and Fisher’s exact test was used for small sample sizes. Analyses were performed using SPSS version 20.0 (IBM Corporation), and a *p-*value <0.05 was considered to be statistically significant.

### Ethical approval

The Ethics committee of The First Affiliated Hospital, College of Medicine, Zhejiang University waived the need for formal ethical approval. No institutional review board approval was necessary, because all the personal information of the patients (such as name, height, weight, etc) was not collected during the investigation and the investigation did not have any impact on the patient management. All patients were informed verbally of our study’s purpose before sending their pathogens to our laboratory for research.

## Results

### Patient demographics and specimen types

A total of 550 isolates were collected and confirmed as *E. coli* by MALDI-TOF. The majority of the isolates were recovered from urine specimens (68.5%), with 8.2% from blood samples, 7.0% from sputum samples, 6.8% from abscesses, 6.0% from secretions (ascites, pleural fluid), and 3.5% from other specimens. Females provided 65% of the isolates. The ages of patients were as follows: ≤17 years, 6.1%; 18–45 years, 39.2%; 46–64 years, 38.3%; and ≥65 years, 16.4%.

### Antimicrobial susceptibility profiles

The results of antimicrobial susceptibility tests for all isolates and ESBL-producing strains are summarised in Table 2. All isolates were susceptible to biapenem, imipenem, and meropenem, whereas 94.1%, 93.6%, and 91.0% of strains were susceptible to fosfomycin, amikacin, and piperacillin-tazobactam, respectively. In total, 249 strains produced ESBLs: 100% of these were resistant to ampicillin, with high resistance also to cefazolin (99.6%, n = 248), ceftriaxone (98.8%, n = 246), ampicillin-sulbactam (61.3%, n = 153), ciprofloxacin (73.5%, n = 183), and levofloxacin (68.8%, n = 171).

**Table 2:** **Results for susceptibility tests and MICs for** ***E. coli*** **strains (n = 550) isolated from 30 county hospitals**

	All isolates (n = 550)				ESBL-positive (phenotype) isolates (n = 249)			
Antibiotic	MIC50 (mg/L)	MIC90 (mg/L)	Susceptible %	Resistant %	MIC50 (mg/L)	MIC90 (mg/L)	Susceptible %	Resistant %
Ampicillin	256	>256	13.6	85.1	>256	>256	0	100
Piperacillin	64	>256	31.8	49.5	256	>256	0	91
Ampicillin-Sulbactam	16	64	35	39.1	32	64	14.7	61.5
Piperacillin-tazobactam	8	16	91	2.1	8	32	86.5	4.5
Cefazolin	32	256	46.7	51.1	256	256	0.4	99.6
Cefuroxime	8	256	50.1	47.8	256	256	0	98.5
Ceftazidime	0.5	32	80.4	18.3	4	64	55.6	40.6
Ceftriaxone	0.25	128	55.4	44.2	64	128	0.7	98.5
Cefepime	1	32	77.7	13.5	8	64	54.5	28.9
Cefoxitin	4	32	76.9	15	8	128	61.8	24.7
Biapenem	0.064	0.125	100	0	0.064	0.125	100	0
Imipenem	0.125	0.25	100	0	0.125	0.25	100	0
Meropenem	0.064	0.125	100	0	0.064	0.125	100	0
Amikacin	2	8	93.6	6.1	2	64	89.1	10.2
Gentamicin	16	128	47.2	51.5	64	256	33.5	66.2
Ciprofloxacin	4	64	46.7	51	32	128	24.8	73.3
Levofloxacin	4	32	49.1	46.7	16	64	26.2	68.9
Fosfomycin	1	32	94.1	5.1	1	128	88.6	9.8

### Genotypic characterisation and geographical distribution of ESBL- and AmpC-producing isolates

**Figure 1:**
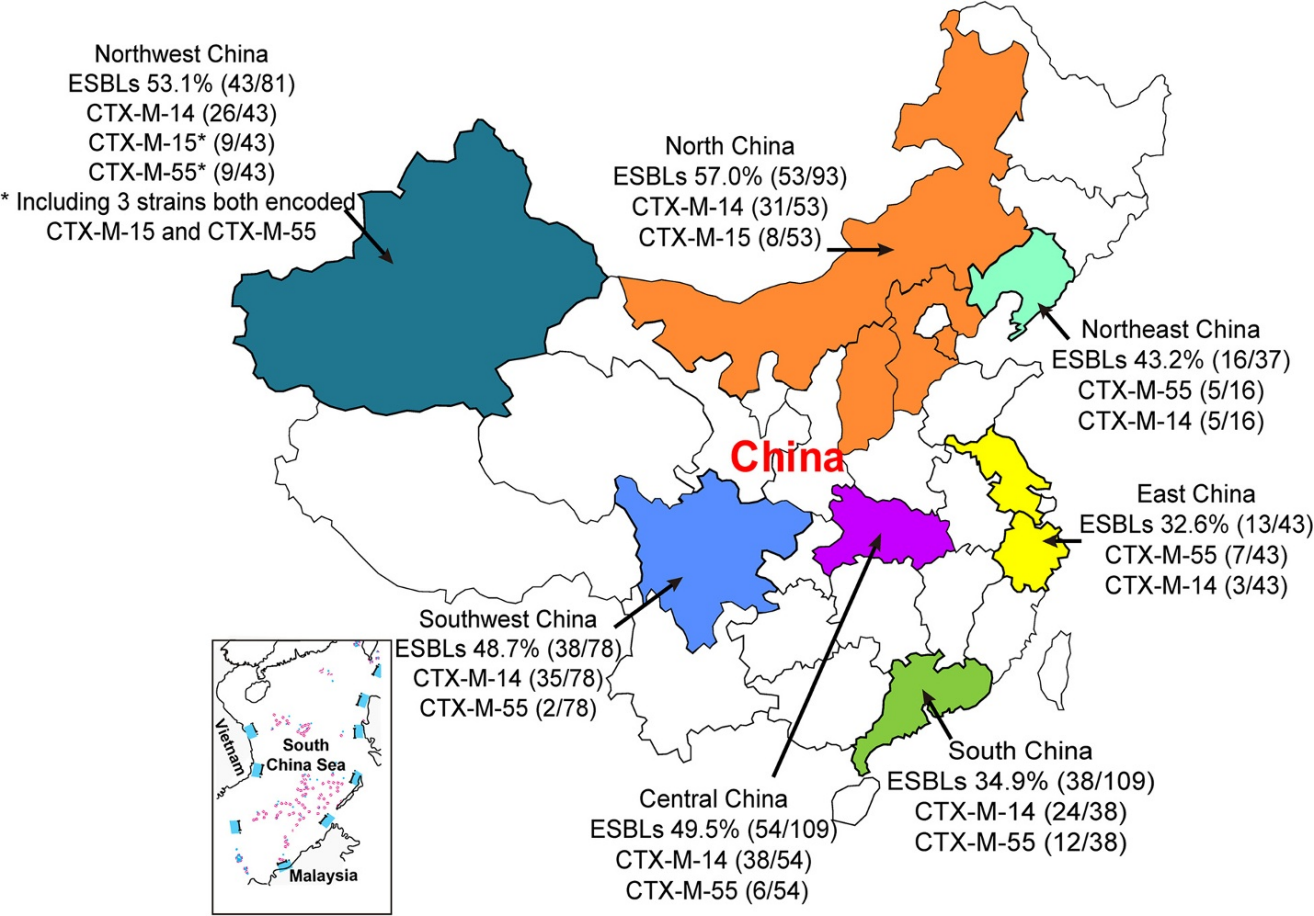
**National wide distribution of clinical isolates and ESBL-producing** ***E. coli.*** Geographical location of seven regions from which strains were collected demonstrated in different colour. The two most dominant genotypes in each region were labelled following the prevalence rate of ESBLs.

PCR was performed on all 550 isolates to determine the presence of ESBL and AmpC. These results and the geographic distributions of the isolates are shown in Table 3. The findings revealed that 256 isolates (46.5%) carried ESBL genes, all of which were the CTX-M type: the CTX-M-9 group (180 isolates) was most abundant, followed by the CTX-M-1 group (91 isolates). The CTX-M-2 and CTX-M-8 groups were not detected. Overall, 12 *bla*_CTX-M_ subtypes were detected: these were *bla*_CTX-M-14_ (164 isolates, 65%, including 17 strains co-existed with other *bla*_ESBL_ or *amp*C genes), which is now the most common ESBL in China, *bla*_CTX-M-55_ (47 isolates, 17.7%, including 6 strains co-existed with *bla*_CTX-M-14_), *bla*_CTX-M-15_ (30 isolates, 11.7%, including 7 strains co-existed with *bla*_CTX-M-14_ or *bla*_CMY-2_ genes), *bla*_CTX-M-3_ (9 isolates, 3.5%, including 3 strains co-existed with other *bla*_ESBL_ or *amp*C genes), *bla*_CTX-M-24_ (9 isolates, 3.5%, including 2 strains co-existed with other *bla*_ESBL_ or *amp*C genes), *bla*_CTX-M-65_ (4 isolates, 1.6%), *bla*_CTX-M-1_ (2 isolates, including 1 strain co-existed with *bla*_CTX-M-14_), *bla*_CTX-M-12_ (1 isolate), *bla*_CTX-M-10_ (1 isolate), *bla*_CTX-M-27_ (1 isolate), *bla*_CTX-M-104_ (1 isolate), and *bla*_CTX-M-9_ (1 isolate). Fifteen isolates encoded ESBLs from both CTX-M-1 and CTX-M-9 groups (Table 3, Figure 1).

**Table 3:** **Geographical distribution of ESBL- and AmpC-producing** ***E. coli*** **isolates in seven regions of China**

ESBL genotype	No. of isolates (prevalence, %)							
	North China ( ***n*** = 93)	Northwest China ( ***n*** = 81)	Northeast China ( ***n*** = 37)	East China ( ***n*** = 43)	South China (n = 109)	Central China (n = 109)	Southwest China (n = 78)	Total ( ***n*** = 550)
CTX-M-1 group	16	14	7	10	14	10	2	73
CTX-M-15	7	6	1	2	3	4		23
CTX-M-55	6	6	5	7	10	5	2	41
CTX-M-3	3	1		1		1		6
CTX-M-101		1						1
CTX-M-12			1					1
CTX-M-1	1							1
CTX-M-9 group	32	22	5	3	23	40	36	161
CTX-M-14	29	19	4	3	22	35	35	147
CTX-M-9		1						1
CTX-M-24		2	1		1	2	1	7
CTX-M-27	1							1
CTX-M-65	2					2		4
CTX-M-104						1		1
CMY-2	1	3	2	1				7
DHA-1	2							2
CTX-M-1+9 groups	1	7	2		2	3		15
CTX-M-14+CTX-M-1	1							1
CTX-M-14+CTX-M-3		1				1		2
CTX-M-14+ CTX-M-55		3			2	1		6
CTX-M-14+ CTX-M-15		3	1			1		5
CTX-M-24+ CTX-M-3			1					1
CTX-M-14+OXA-10	1							1
CTX-M-3+ DHA			1					1
CTX-M-14+DHA	2							2
CTX-M-15+CMY-2	1						1	2
CTX-M-24+CMY-2	0		1					1
Total^a^	53 (57.0)	43 (53.1)	16 (43.2)	13 (30.2)	38 (34.9)	54 (49.5)	39 (50.0)	256 (46.5)

^a^The number exclude the strains that only encoded *amp*C gene.

The prevalence of ESBL-producing *E. coli* strains varied significantly (*p* < 0.01). among the different regions of China, with the highest proportion (57.0%) in North China and the lowest proportion (30.2%) in East China (EC). The prevalence of ESBL-producing isolates significantly differed among different hospitals within South China (SC1–SC5) (*p* < 0.05), the prevalence of ESBL-producing isolates was high in one hospital (SC4; 72.7%; 8/11 strains) and low in another hospital (SC1; 16.7%; 6/36 strains), there was a similar situation within different hospitals in Northwest China (NW1–NW4) (*p* < 0.05). There were no statistical differences in the prevalence of ESBL-producing *E. coli* strains between different hospitals in the remaining five regions.

CTX-M-14 (CTX-M-9 group) was the most common ESBL genotype detected in this study; however, the results suggest that CTX-M-55 (CTX-M-1 group) is also becoming prevalent, with the ratio of the number of CTX-M-55-positive isolates to the number of ESBL-positive isolates being 5:8 in SC4, 4:8 in SC5, 6:9 in EC2, and 4:8 in NE1. By contrast, CTX-M-15 was the predominant genotype in EC3 and NW2.

There was no statistical difference (*p* = 0.6) between *E. coli* strains isolated from urinary tract infections (n = 324, 46.0% harboured genes for ESBLs), and those isolated from blood (n = 44, 40.9% harboured genes for ESBLs).

In total, ten isolates encoded CMY-2 *ampC* genes and five encoded DHA-1 *ampC* genes; three of the CMY-2 *ampC* genes and three DHA-1 *ampC* genes co-existed with *bla*_CTX-M_.

### Characterisation of non-ESBL β-lactamase genes

Genes for the broad-spectrum β-lactamases TEM-1 (347 strains), OXA-1 (35), and SHV-11 (5) were most prevalent among isolates, and were detected either alone or co-existing with other enzymes. One strain contained the gene for the inhibitor-resistant β-lactamase TEM-30 (and CTX-M-55) and showed no ESBL activity. One strain contained genes for both CTX-M-14 and OXA-10. We believe this is the first reported detection of OXA-10 in *E. coli* in China. The KPC was not detected in this study.

### Molecular typing of ESBL-producing E. coli isolates

**Figure 2:**
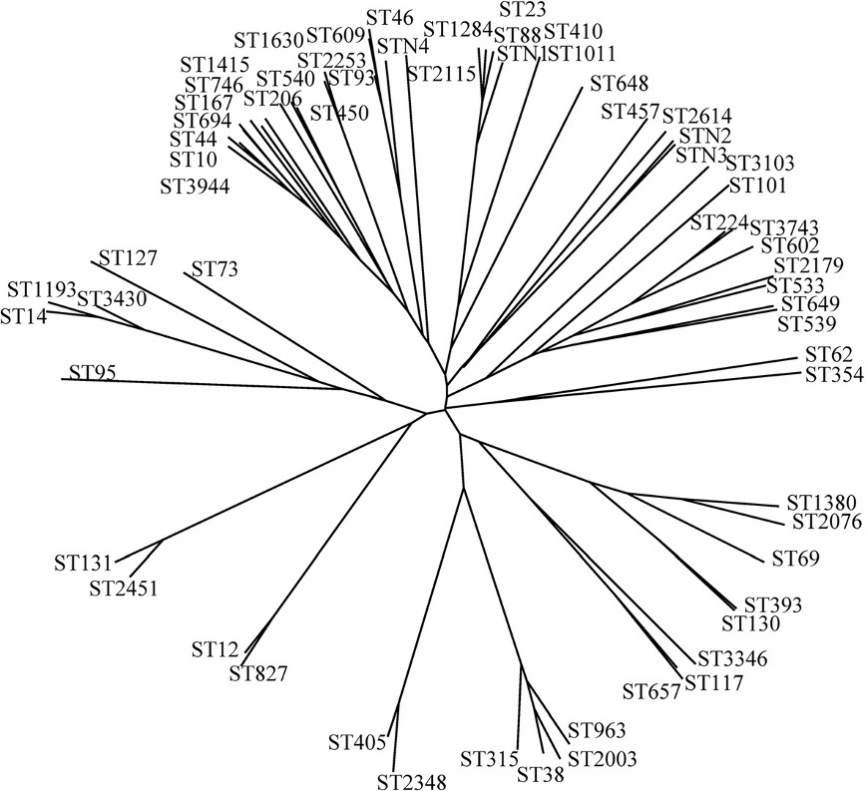
**Phylogenetic tree of STs detected in this study using Phylodendron.** This tool uses the PHYLIP suite of programs to generate trees from allelic profile data. Phylogenetic tree generated in this study indicated there was no concentrated distribution of STs in ESBL-producing genotypes.

MLST revealed 64 different STs, and nine new STs that are not registered in the *E. coli* MLST database. ST131 was the most abundant type (27 isolates, 12.7%), followed by ST69 (14 isolates, 6.6%), ST405 (14 isolates, 6.6%), and ST38 (12 isolates, 5.6%). The sources for the ST131 isolates were distributed across 14 hospitals from seven regions. Among the ST131 isolates, 18 were positive for CTX-M-14, five were positive for CTX-M-55, three were positive for CTX-M-15, and one was positive for both CTX-M-14 and CTX-M-3. Three Northwest hospitals provided 66.7% of the ST38 strains (n = 12). These three hospitals are located far from each other in Xinjiang province. One hospital accounted for 50% (4/8 isolates) of ESBL-producing strains. ST38 was the dominant type in these three hospitals, but was not found in the one hospital in Xinjiang. A phylogenetic tree of STs detected in this study using a Phylodendron is shown in Figure 2.

## Discussion

The findings of this study reveal, for the first time, the high prevalence of ESBL-producing *E. coli* associated with community-onset infections in county hospitals across China. The results indicate that 46.5% of clinical *E. coli* isolates carried genes for ESBLs, and that this figure varied significantly across the different regions, ranging 30.2% to 57.0%. In particular, the proportion of isolates that carried ESBL genes was 50% or greater in the North, Northwest, and Southwest regions of China. PCR amplification revealed a high diversity of CTX-M-type ESBLs in China, with 12 subtypes detected. The results showed that CTX-M-14 was the most abundant of those CTX-M enzymes detected, and that it is prevalent across China. This is supported by previous studies that have reported the prevalence of ESBLs in isolates from both community-associated and hospital-acquired infections in Chinese tertiary hospitals, and also in healthy people from large cities [7],[8],[32].

A developing characteristic of ESBL epidemiology in several hospitals is the emergence of CTX-M-55 as a dominant genotype. This is the first study to report that the abundance of clinical isolates in China containing CTX-M-55 have exceeded those with CTX-M-15 as the second most common genotype. In 2009, CTX-M-55 was detected in only two of 49 CTX-M-1 group *E. coli* strains in Guangzhou Province in South China [33]. Following this report, there were limited investigations into the presence of CTX-M-55 in Chinese clinical isolates. However, Hu *et al.* recently reported that eight CTX-M-55 strains were detected from 17 CTX-M-1 group *E. coli* isolates from the faeces of healthy humans in Hangzhou (Zhejiang Province) [32].

CTX-M-15 differs from CTX-M-55 by a single amino acid substitution of Ala-77-Val. CTX-M-55 was first detected in clinical isolates of *E. coli* and *K. pneumonia* from Thailand in 2005 [34], and was subsequently found in *Salmonella* spp. in China, the United States, Korea, and Switzerland [35]-[37]. Although CTX-M-55 is widely distributed in *E. coli* strains isolated from pets and food-producing animals, its range currently appears restricted to specific geographic regions, mainly in Asia [15],[34],[38]-[41]. Two previous studies of the epidemiology of ESBL-producing *E. coli* strains in food-producing animals in China reported that CTX-M-55 was the second (26.1%, 29/111) and third (18.5%, 10/54) most common genotype, both following CTX-M-14 [39],[42]. A further study showed that CTX-M-55 was the second most common (25%, 27/108 strains) genotype detected among ESBL-producing *E. coli* strains isolated from healthy ducks and environmental samples from a duck farm in South China [40]. These findings suggest that CTX-M-55 has already transferred from animals to humans, and spread among both healthy individuals and patients in China, and that this particular β-lactamase may be displacing *bla*_CTX-M-14_ as the most common genotype in some regions.

Drug resistance in *E. coli* can be transferred horizontally between strains that are common to food-producing animals and humans. *E. coli* can also transfer resistance genes to bacteria that are part of the commensal intestinal flora, and it may serve as an important reservoir for these transmissible traits [43]-[47]. Winokur *et al*. reported plasmid transfer of β-lactamase genes between *Salmonella* and *E. coli,* and suggested that they may have been transmitted between food-producing animals and humans [44]. Fey *et al.* reported the acquisition of ceftriaxone-resistant *Salmonella enterica* serotype typhimurium by a 12-year-old boy from cattle [45]. This finding highlights the potential threat to public health through the transfer of highly resistant bacterial strains from livestock to humans, particularly farmers, ranchers, and animal handlers [45]. Findings from this study, and others, suggest that CTX-M-55 can be transmitted from animals to humans. Most outpatients in county hospitals live in rural areas and are likely to have contact with infected food-producing animals and farm sewage. Further investigations are needed to test this hypothesis and resolve the mechanism by which *bla*CTX-M-55 transfers to, and exists in, humans.

In this study, 64 different STs (including nine new STs) were detected among 213 ESBL-producing *E. coli* isolates, and ST131 was the most abundant type (12.7%). These findings are similar to those from a tertiary hospital investigation, which reported that 9.6% of 94 *bla*_CTX-M-14_- and *bla*_CTX-M-15_-containing *E. coli* strains belonged to ST131 [48]. An investigation of ESBL-producing *E. coli* isolated from river samples and the faeces of healthy humans found that 14.4% (20/139) of isolates belonged to ST131 [32]. The findings indicate that no particular ST of *E. coli* is predominant in China, unlike European and North American countries where there is a higher prevalence of ST131 [6]. For example, 53% of ESBL-producing *E. coli* isolates belonged to ST131 in the United States [5]. A study from Calgary for the period 2000–2007 revealed that *E. coli* clone ST131, which produces CTX-M-15, was an important cause of community-onset bacteraemia, with its incidence increasing from 6% (1/18) in 2000–2003 to 41% (20/49) in 2004–2007 [49]. In one region of the United Kingdom, 64% of community-acquired cefpodoxime-resistant *E. coli* infections were caused by ST131 [50]. Similarly, data from Belgium covering the period 2006–2007 shows that 62% of CTX-M-15-carrying *E. coli* causing community-acquired infections belonged to ST131.[51] In France, 25% of community-onset ESBL-producing *E. coli* infections belonged to ST131 [13].

No dominant ST type was detected in any region; however, in Northwest China, 19.5% (8/41) of isolates belonged to ST38 (detected in three of four hospitals). These four hospitals are all located in Xinjiang province, and are far away from each other. Results indicated that 164 *bla*_CTX-M-14_-containing strains clustered into 40 different STs, and 47 *bla*_CTX-M-55_-containing strains were scattered between 22 different STs. There was no concentrated distribution of STs in these two, or any other, ESBL-producing genotypes (Figure 2). The results indicate no evolutionary convergent relationships among ESBL-producing *E. coli* in Chinese hospitals, and suggest that the clonal dissemination of ESBL-producing strains is complicated in China and requires further investigation.

This study provides the first report of an OXA-10-like β-lactamase sequence detected in a member of the *Enterobacteriaceae* in China, although it was previously reported in *Pseudomonas aeruginosa* [52]. OXA-10 has also been recorded in multi-drug-resistant clinical isolates of *E. coli*, *K. pneumonia,* and *Proteus. mirabilis* [53]-[55]. OXA-10 is a class D broad-spectrum β-lactamase that hydrolyses oxacillin, methicillin, and cloxacillin, and is inhibited by sodium chloride and clavulanic acid [56]. This β-lactamase was first detected in *P. aeruginosa* in 1988 [56], and has a similar function to OXA-2 as the parent of OXA-type ESBL, the extended-spectrum variant was continually found in *P. aeruginosa* during the past two decades, although it has not been detected in *Enterobacteriaceae* [57],[58]. The detection of OXA-10 in the *Enterobacteriaceae* reported here, and in other studies, suggests further investigations are needed into the origin of this *P. aeruginosa*-derived β-lactamase and its transmission.

## Conclusions

In summary, this study reports high rates of ESBL-producing isolates of *E. coli* community-associated infections in county hospitals in China; although the prevalence was lower than in tertiary hospitals. The CTX-M ESBL was the dominant enzyme, and CTX-M-14 was the major molecular type detected in community-onset infections. A diverse range of STs was detected, and no evolutionary convergent relationship among ESBL-producing *E. coli* was found. We also report the detection of the OXA-10 type of ESBL in clinical isolates of *E. coli.* These evolutionary characteristics of ESBL epidemiology in clinical strains of *E. coli* may complicate the future management of antibiotic resistance in China if no effective measures are taken. Further investigations are needed to resolve the mechanisms underlying the increasing incidences of CTX-M-55 and the emergence of OXA-10 in strains of *E. coli* in county hospitals.

## Additional file

## Electronic supplementary material

Additional file 1: List of the participant hospitals. The list and the geographic of 30 county hospitals in this study. (DOCX 18 KB)

Below are the links to the authors’ original submitted files for images.

Authors’ original file for figure 1

Authors’ original file for figure 2
