# Supplementary material for: Nationwide high prevalence of CTX-M and an increase of CTX-M-55 in Escherichia coli isolated from patients with community-onset infections in Chinese county hospitals
Source: BMC Infect Dis. 2014 Dec 3;14:659. doi: 10.1186/s12879-014-0659-0 (PMC4265337; doi:10.1186/s12879-014-0659-0)
Supplement: Supplementary file 1 — Additional file 1: List of the participant hospitals. The list and the geographic of 30 county hospitals in this study. (DOCX 18 KB) [file 12879_2014_659_MOESM1_ESM.docx]

| Region | Hospital name | serial number |
| --- | --- | --- |
| North China | Second People's Hospital of Hengshui | NC1 |
| North China | Qinhuangdao military hospital | NC2 |
| North China | Linhe district People's Hospital of Inner Mongolia | NC3 |
| North China | Zalantun People's Hospital Inner Mongolia | NC4 |
| North China | Jishan County People's Hospital of Shanxi Province | NC5 |
| North China | Tianjin Beichen Hospital | NC6 |
| Northwest China | First People's Hospital in Urumqi, Xinjiang | NW1 |
| Northwest China | Hetian People's Hospital | NW2 |
| Northwest China | Ili Kazak Autonomous Prefecture Chinese Medicine Hospital | NW3 |
| Northwest China | Second Division hospital of Xinjiang Korla | NW4 |
| East China | Taixing People's Hospital | EC1 |
| East China | Shangyu People's Hospital | EC2 |
| South China | Shenzhen Baoan People's Hospital | SC1 |
| South China | Longgang Central Hospital | SC2 |
| South China | Panyu District People's Hospital of Guangzhou | SC3 |
| South China | Foshan Shunde Longjiang Hospital | SC4 |
| South China | Dongguan Hengli Hospital | SC5 |
| Central China | Second People's Hospital of Jingzhou | MS1 |
| Central China | Yiling Hospital of Yichang | MS2 |
| Central China | Zhijiang People's Hospital of Hubei | MS3 |
| Central China | Yingshan People's Hospital of Hubei | MS4 |
| Central China | Jianli County People's Hospital of Hubei | MS5 |
| Northeast China | Jinzhou Hospital of Dalian | NE1 |
| Northeast China | Shenyang Women and Children's Hospital | NE3 |
| Northeast China | Yingkou Development Zone Hospital | NE4 |
| Southwest China | Shifang People's Hospital of Sichuan | SW1 |
| Southwest China | Jiangyou People's Hospital of Sichuan | SW2 |
| Southwest China | Mianyang People's Hospital of Sichuan | SW3 |
| Southwest China | Renshou People's Hospital of Sichuan | SW4 |
| Southwest China | Suining First People's Hospital of Sichuan | SW5 |
