# Supplementary figures and images for: Nationwide high prevalence of CTX-M and an increase of CTX-M-55 in Escherichia coli isolated from patients with community-onset infections in Chinese county hospitals
Source: BMC Infect Dis. 2014 Dec 3;14:659. doi: 10.1186/s12879-014-0659-0 (PMC4265337; doi:10.1186/s12879-014-0659-0)

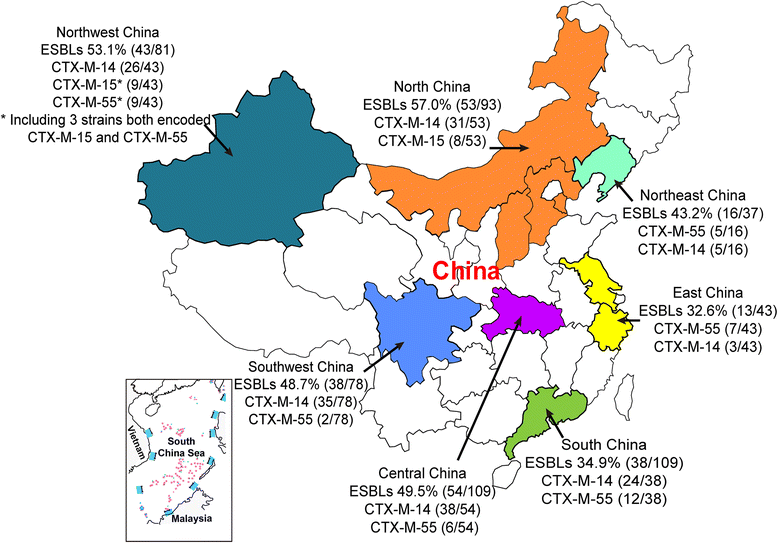

Supplement: Supplementary file 2 — Authors’ original file for figure 1 [file 12879_2014_659_MOESM2_ESM.gif]

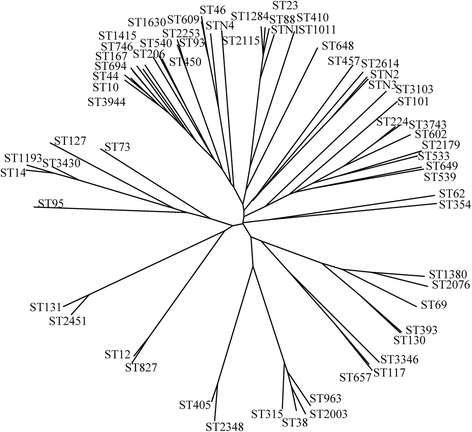

Supplement: Supplementary file 3 — Authors’ original file for figure 2 [file 12879_2014_659_MOESM3_ESM.gif]
